# Moisture Effect on Particulate Matter Filtration Performance using Electro-Spun Nanofibers including Density Functional Theory Analysis

**DOI:** 10.1038/s41598-019-43127-4

**Published:** 2019-05-07

**Authors:** Han-Jung Kim, Seon Joo Park, Dong-Ik Kim, Sanghyuck Lee, Oh Seok Kwon, Il Ku Kim

**Affiliations:** 1 Advanced Materials Component Research Center, Gumi Electronics & Information Technology Research Institute (GERI), Gumi, 39171 South Korea; 2 Infectious Disease Research Center, Korea Research Institute of Bioscience and Biotechnology (KRIBB), Daejeon, 34141 South Korea; 3 Center for Integrated Smart Sensors (CISS), Korea Advanced Institute of Science and Technology (KAIST), Daejeon, 34141 South Korea; 4 Nanobiotechnology and Bioinformatics (Major), University of Science & Technology (UST), 125 Gwahak-ro, Yuseong-gu, Daejeon, 34141 South Korea; 5 Institute of Integrated and Intelligent Systems, Griffith University, Brisbane, Queensland 4111 Australia; 6 Brain Gear Incorporation, 409 Expo-ro, Yuseong-gu, Daejeon, 34051 South Korea

**Keywords:** Environmental sciences, Theory and computation, Nanoscience and technology

## Abstract

In this study, we use density functional theory (DFT) calculations to investigate the effect of moisture on the performance of three types of nanofiber (NF)-based air-filter media prepared by electrospinning polyvinyl alcohol, polyvinylidene fluoride, and polyacrylonitrile (PAN). Based on the DFT calculations of the intermolecular interactions between the NF-based filter media and water molecules, the PAN-NF filter is expected to exhibit the best performance in the wet state. Experiment studies also successfully demonstrate that the PAN-NF filter medium has better performance in the filtration of particulate matter (PM) than a commercial semi-high efficiency particulate air filter under wet conditions, and these results are in good agreement with the DFT calculation. The PAN-NF filter shows better performance because of its hydrophilic nature and the relatively low thickness the filter medium that allowed fast recovery of its PM-filtration performance.

## Introduction

The pollution of air by particulate-matter (PM) is a serious concern in developing countries because of rapid industrialization and population growth^1–3^. Coarse PM measuring less than 10 μm in diameter, also known as PM_10_, originates primarily from road dust, agricultural dust, construction sites, mining operations, and similar activities. Fine PM measuring less than 2.5 μm in diameter, known as PM_2.5_, is mainly caused by burning fuels and combustion. When PMs are inhaled by the human body, they can cause serious problems in the heart and lungs^4,5^. Therefore, PM was recently designated as a Group 1 carcinogen by the International Agency for Research on Cancer (IARC) of the World Health Organization (WHO). Guidelines were established to limit average exposure to a maximum of 10 μg/m^3^ of PM_2.5_ per year, and a maximum of 25 μg/m^3^ over a 24-h period^6,7^. Consequently, many studies have been conducted on the capture and removal of PM such as electric precipitators^8^, catalytic filters^9^, cyclonic separators^10^, wet scrubbers^11^, and fabric filters^12–15^. Among them, nanofiber (NF)-based air-purification (or filtration) technology has attracted particular interest in recent years owing to its high performance (low pressure drop, high filtration efficiency, and dust-holding capacity), durability (service life, high-temperature stability, and flexibility), and high productivity (large-area production and mass production)^16,17^. To support the technology, different types of air filters have been developed using NFs of polymers such as polyvinyl alcohol (PVA), polyvinylidene fluoride (PVDF), and polyacrylonitrile (PAN)^16–18^. To date, much of investigations (structural and chemical properties; diameter, packing density, pore size, specific surface area, etc.) has been carried out to improve of NF performances in terms of changing fabrication conditions^16–18^. However, studies of polymer NF-based air filters are just considered on less moisture ambient^19–21^. In spite of practical use on less moisture ambient, a great demand is emerging for development of washable filters that can be reused after washing^12,22,23^. Therefore, it is inevitable to investigate the filtration performance and durability of NF-based air filter media in high-humidity environments before they can be used.

Our motivation of this study is establishing foundation of the development of NF-based air filter media that can effectively capture PM even in high moisture environments. We fabricated three different types of polymer-NF-based air filter media by electrospinning method and investigated the changes in filtration performance in the wet state. Furthermore, the intermolecular interactions between the NF-based filter media and water molecules were analyzed by density function theory (DFT) calculations and compared to the experimental results. Compared with the commercial semi-high-efficiency particulate air (semi-HEPA) filter media, the NF-based filter media were observed to show relatively fast performance recovery. In particular, the PAN-NF filter medium showed superior performance in recovery of its filtration capability.

## Results and Discussion

**Figure 1:**
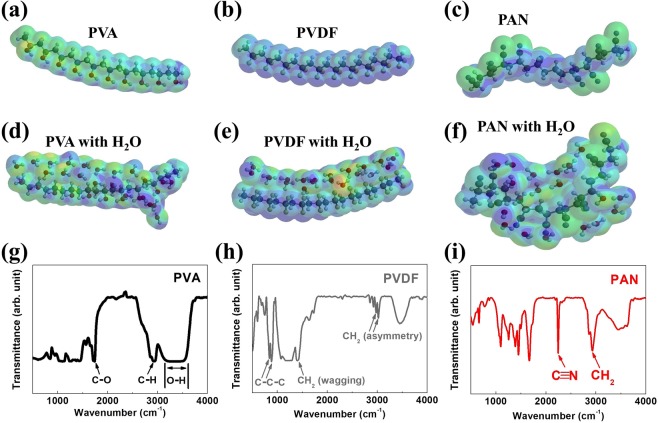
DFT calculation results for (**a**) 2 nm linear PVA molecule without H_2_O, (**b**) 2 nm linear PVDF molecule without H_2_O, (**c**) 2 nm twisted PAN molecule without H_2_O, (**d**) 2 nm PVA molecule with 20 H_2_O molecules, (**e**) 2 nm PVDF molecule with 20 H_2_O molecules, and (**f**) 2 nm PAN molecule with 20 H_2_O molecules. FTIR results for (**g**) PVA-NF filter medium, (**h**) PVDF-NF filter medium, and (**i**) PAN-NF filter medium.

Figure 1(a–f) show the DFT calculation results for PVA, PVDF, and PAN molecules (length: 2 nm) with or without H_2_O molecules to predict the effect of moisture. The geometry and energies of PVA, PVDF, and PAN were optimized using the B3LYP/6–31 G* method^24–26^. The results of single- and small-molecule DFT calculations of all NFs are also provided in Fig. S1 (see Supporting Information). The results of dipole-moment (DPM) calculations are summarized in Table 1. It should be noted that our calculated DPM of a single molecule is quite similar to the recently reported value^27^. As shown in Fig. 1(a,b), the PVA and PVDF molecules appear to have a linear shape by increasing of molecular weight. However, as shown in Fig. 1(c), the external shape of the PAN molecule becomes twisted as the molecular weight increases. A comparison of the molecular shapes and DPMs shows that the increase in DPM of a linear shape NF molecule (1.67 to 23.62 D for PVA; 2.06 to 16.8 D for PVDF) is greater than a twisted NF molecule (3.91 to 9.43 D for PAN); this difference originates from the molecular structure. Interestingly, the addition of a H_2_O molecule in the DFT calculation shows dramatic differences between PVA, PVDF, and PAN. For PVA and PVDF, the H_2_O molecules are not fully distributed in the PVA and PVDF structures, and the distance between H_2_O and NFs are 3.6 Å for PVA and 3.4–4.1 Å for PVDF. In PAN, however, the H_2_O molecules are fully distributed and having an average distance of 3.0 Å (Fig. S1). This would be a strong supporting point in water droplet experimental results and will be discussed further. From Table 1, the increase in DPM resulting from the addition of a H_2_O molecule varies widely between PVA, PVDF, and PAN. For single molecules (PVA, PVDF, and PAN) with a single H_2_O molecule, PAN shows the highest DPM of with 6.02 D, while the DPMs of PVA and PVDF is remaining in the range of 2.45–2.76 D. However, when the molecular weight and number of H_2_O molecules are increased for PVA, PVDF, and PAN, the increase in DPM follows the same trend as that shown in the DFT calculations without H_2_O molecules. However, regardless of the presence or absence of H_2_O molecules, the DPMs of PVA and PVDF are still higher than that of PAN, which suggests that the effect of water or moisture in PVA and PVDF is insignificant. However, in the case of PAN, the values of DPM decreases from 9.61 to 6.67 D (small-molecule PAN without and with H_2_O) and from 9.43 to 6.22 D (2 nm PAN without and with H_2_O). Therefore, this also suggests that water or moisture can be uniformly distributed in the PAN system by absorption even if it consists of small molecules or has higher molecular weight.

**Table 1:** Dipole moments (DPMs) of PVA, PVDF, and PAN molecules with or without H2O for single molecules, small molecules, and 2 nm-long molecules.

	PVA	PVDF	PAN
Single molecule	1.67 D	2.06 D	3.91 D
Small molecule	6.09 D	5.41 D	9.61 D
2 nm molecule	23.62 D	16.80 D	9.43 D
Single molecule + 1 H_2_O molecule	2.45 D	2.76 D	6.02 D
Small molecule + 4 H_2_O molecules	4.29 D	11.07 D	6.67 D
2 nm molecule + 20 H_2_O molecules	19.55 D	15.40 D	6.22 D

Figure 1(g–i) show the Fourier transform infrared (FTIR) analysis results of the chemical composition of PVA-NF, PVDF-NF, and PAN-NF air-filter media, respectively. In Fig. 1(g), the peak corresponding to O–H stretching lies between 3,200 and 3,550 cm^–1^, the signal corresponding to the C–H alkyl group lies between 2,800 and 3,000 cm^−1^, and the peak at 1,737 cm^−1^ is attributed to C–O stretching^28^. In Fig. 1h, the two peaks at 2,977 and 3,022 cm^−1^ are related to the CH_2_ asymmetric and symmetric vibration, while the peak at 1,404 cm^−1^ is attributed to CH_2_ wagging vibration; the peaks at 840 and 882 cm^−1^ are attributed to C–C–C asymmetrical stretching vibration and C–F stretching vibration, respectively^29^. Finally, in Fig. 1(i), the peak at 2,930 cm^−1^ corresponds to CH_2_ vibration of the hydrocarbon, and the peak at 2,245 cm^−1^ corresponds to C≡N stretching of the acrylonitrile unit in the polymer chain^30^. From these experimental results, it has confirmed that the three types of filter media were produced as intended.

**Figure 2:**
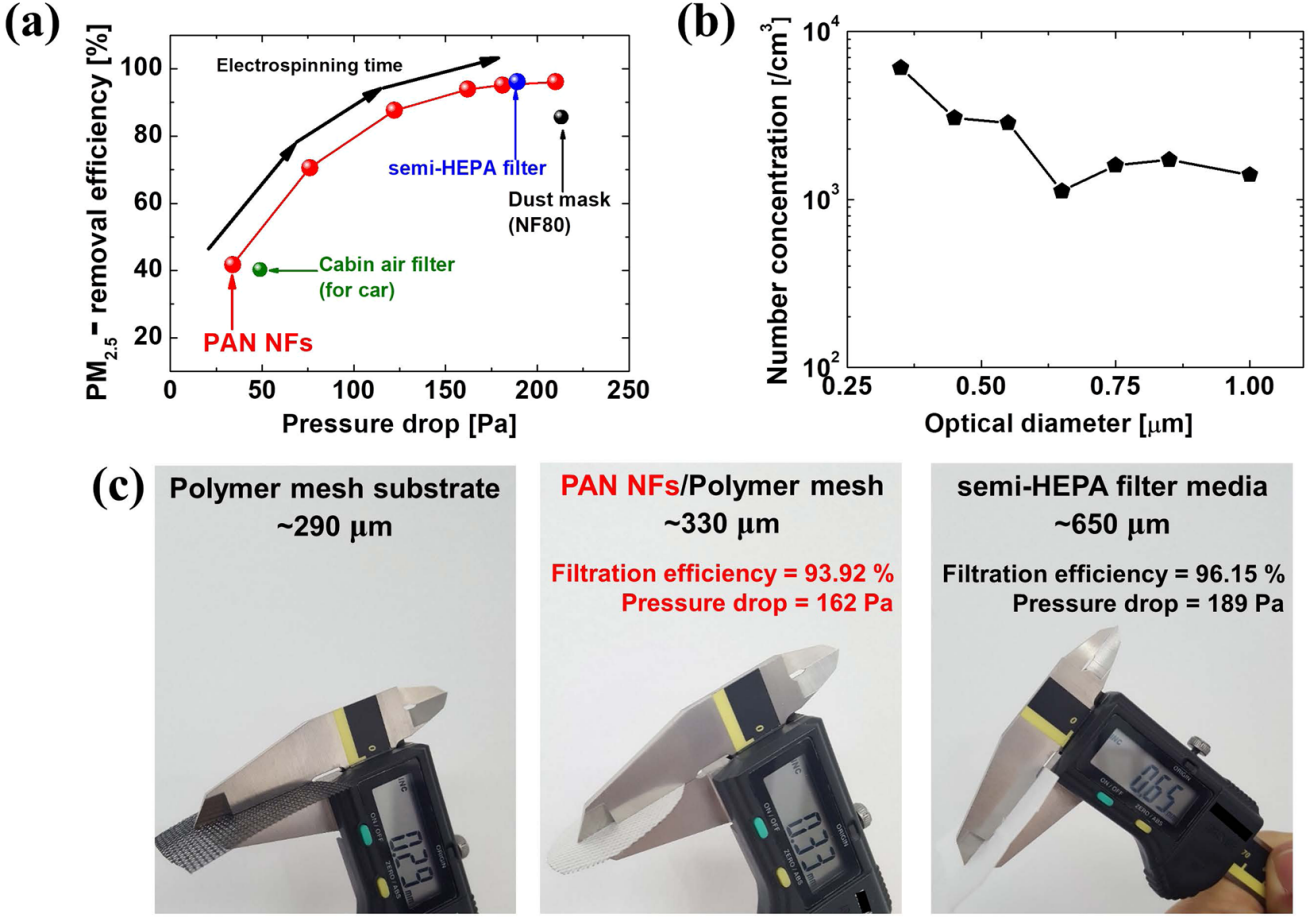
(**a**) Comparison of PM_2.5_-removal efficiency and pressure drop across PAN-NF filter medium and commercial filters (semi-HEPA filter, dust mask, and car cabin filter). (b) Size distribution of incense smoke particles as PM_2.5_ source. (**c**) Comparison of the thickness of PAN-NF filter and semi-HEPA filter.

Figure 2(a) shows a comparison of the PM_2.5_-removal efficiency and pressure drop of the PAN-NF filter medium and three commercial air filter media (car cabin filter, semi-HEPA filter, and dust mask) in a low-moisture ambient. Fig. S2 shows a schematic of the electrospinning process and a photo of a fabricated PAN NF-based filter medium. It should be noted that the PM_2.5_-removal efficiency is increased as the thickness of the NF-based filter medium increasing. In general, it is widely accepted that a filter’s PM-filtration performance can be easily improved by increasing of thickness of the NF-based filter medium or by reducing the pore size of the NF-based filter medium. In the experiment, smoke was generated when burning incense was used as the PM source. The smoke particles varied from 0.3 to 10.0 μm in diameter (Fig. 2(b)), but most of them were in the range of <1.0 µm and were thus categorized as PM_2.5_. In general, it is widely accepted that a filter’s PM-filtration performance can be easily improved by increasing the thickness of the NF-based filter medium^27,31^. In our study, the thickness of the electro-spun NFs on the polymer mesh substrate was controlled by extending the electrospinning duration and monitoring the pressure drop as an indicator of the PM-filtration performance. More importantly, the thickness of the NF-based filter medium is relatively thin compared to that of a conventional filter medium^27,31,32^. In Fig. 2(c), the thickness of the PAN NF filter medium (including the polymer mesh substrate) was about 330 μm, and it showed superior performance (PM_2.5_-removal efficiency of 93.92% and pressure drop of 162 Pa) that was comparable to that of the commercial semi-HEPA filter medium. It is important to note that this performance was attained with a relatively thin PAN NF-based filter medium, whose thickness is 49.2% that of the commercial semi-HEPA filter medium. Therefore, it can assumed that there was a tradeoff between the pressure drop and NF-based filter medium thickness. The specific surface area, thickness, and fiber diameter of a filter medium is an important factor of its filtration performance, dust-holding capacity, and durability^16,31,32^. To verify this, the specific surface area of all filter media was measured by the Brunauer–Emmett–Teller (BET) method. Table 2 shows the measured specific surface area, average fiber diameter, thickness, pressure drop, and PM_2.5_-removal efficiency of a commercial semi-HEPA filter and three different types of NF-based filter media (PVA, PAN, and PVDF). The specific surface area of NF-based filter medium was at least seven times larger than that of the microfiber-based semi-HEPA filter. This implies that the PAN-NF filter medium had higher dust-holding capacity than the commercial semi-HEPA filter medium. In addition, scanning electron microscopy (SEM) images of all of the fiber-based filter media are shown in Fig. S3 for comparison.

**Table 2:** Measured average fiber diameter, specific surface area, thickness, pressure drop, PM_2.5_-removal efficiency, and quality factor (QF) of a commercial semi-HEPA filter and three different types (PVA, PVDF, and PAN) of NF-based filters.

Filter medium	Mean fiber diameter [nm]	Specific surface area [m^2^/g]	Thickness [µm]	Pressure drop [Pa]	PM_2.5_-removal efficiency [%]	Quality factor, QF [Pa^−1^]
PVA	~210	25.4	52	175	70.81	0.007040
PVDF	~590	8.5	43	145	83.01	0.012400
PAN	~500	10.1	40	162	93.02	0.017285
Semi-HEPA	~15,000	1.1	650	189	96.15	0.017233

**Figure 3:**
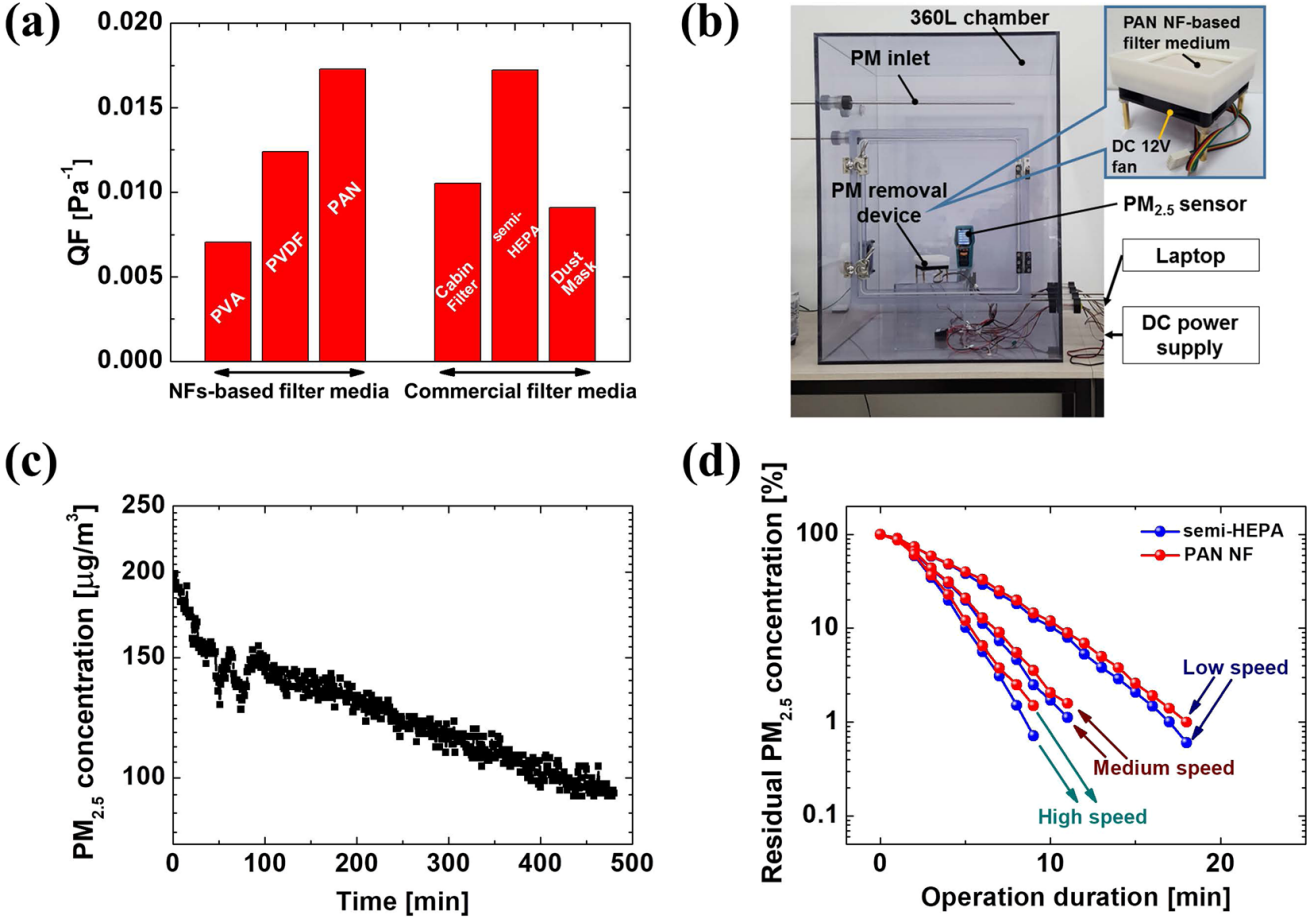
(**a**) Calculation results of quality factor (QF) for all air filters. (**b**) Photo of PM-removal test chamber and (inset) filtration device. (**c**) Change in PM_2.5_ concentration in a closed chamber without a PM-removal device. (**d**) Comparison of the PM_2.5_-removal performance of a PAN-NF-based filter and a semi-HEPA filter.

The figure of merit of the filtration performance of a fibrous air filter medium is measured by the quality factor (QF):

1 $${\rm{QF}}=-\,\mathrm{ln}\,P/{\rm{\Delta }}p,$$

where *P* is the ratio of the penetration rate of particles (or particulate pollutants), and ∆*p* is the pressure drop across the filter medium^27,33^. Here, *P* is equal to 1-*E*, where *E* is the total filtration efficiency of the filter media, while a higher QF indicates better performance of the filter medium. Figure 3(a) shows a comparison of the QF values of three kinds of NF-based filter media (PVA, PVDF, and PAN) and three kinds of commercial filter media (cabin filter, semi-HEPA filter, and dust mask). The calculated QF of the PAN-NF filter medium (0.017285) is higher than the calculated values for the other NF-based filter media (0.00704 for PVA; 0.0124 for PVDF). Based on the molecular structural analysis and DFT calculations for the NF-based filter media, it can be predicted that the PAN has a more close-packed structure than PVA or PVDF (Fig. 1(f)). As shown in Fig. 1(c) and Table 1, PAN is also expected to have higher QF because of its twisted structure and steady-state DPM values from the DFT calculations. The QF of the PAN-NF filter medium is also slightly higher than that of the semi-HEPA filter medium (QF = 0.017233).

To demonstrate the practical use of NF-based filter media, we constructed a closed chamber with a volume of 360 L (Fig. 3(b)) and a PM-removal device (for PM_2.5_) that employed an electro-spun NF-based filter medium with a small direct current (DC) fan (CT-8015H12BF, COOLERTEC, China), as shown in the inset of Fig. 3(b). Before beginning the PM_2.5_-removal test, PM_2.5_ (with a high concentration of about 200 μg/m^3^) was supplied into the chamber without any PM-removal device, and the change in PM_2.5_ concentration in the chamber was observed over time. Figure 3(c) shows that the PM_2.5_ concentration gradually decreased for 8 h from 200 to 95 μg/m^3^. Figure 3(d) shows the PM-capturing ability of the commercial semi-HEPA and PAN -NF filter media in the contaminated chamber with high PM_2.5_ concentrations (about 1,000 μg/m^3^) with a PM-removal device. For comparison, the same voltage was applied to the PM-removal device’s fan at DC 6 (for low speed), 9 (for medium speed), and 12 V (for high speed), respectively. As a result, the PAN-NF filter medium showed comparable performance (air filtration rate) with the commercial semi-HEPA filter medium. It is important to point out that the comparable performances were also due to the inherent characteristics of the PAN-NF filter medium with a relatively low pressure drop (pressure drop of 162 Pa) compared to the commercial semi-HEPA filter medium (pressure drop of 189 Pa). This indicates that there was better air flow through the PAN-NF filter medium during the PM_2.5_-capture process.

**Figure 4:**
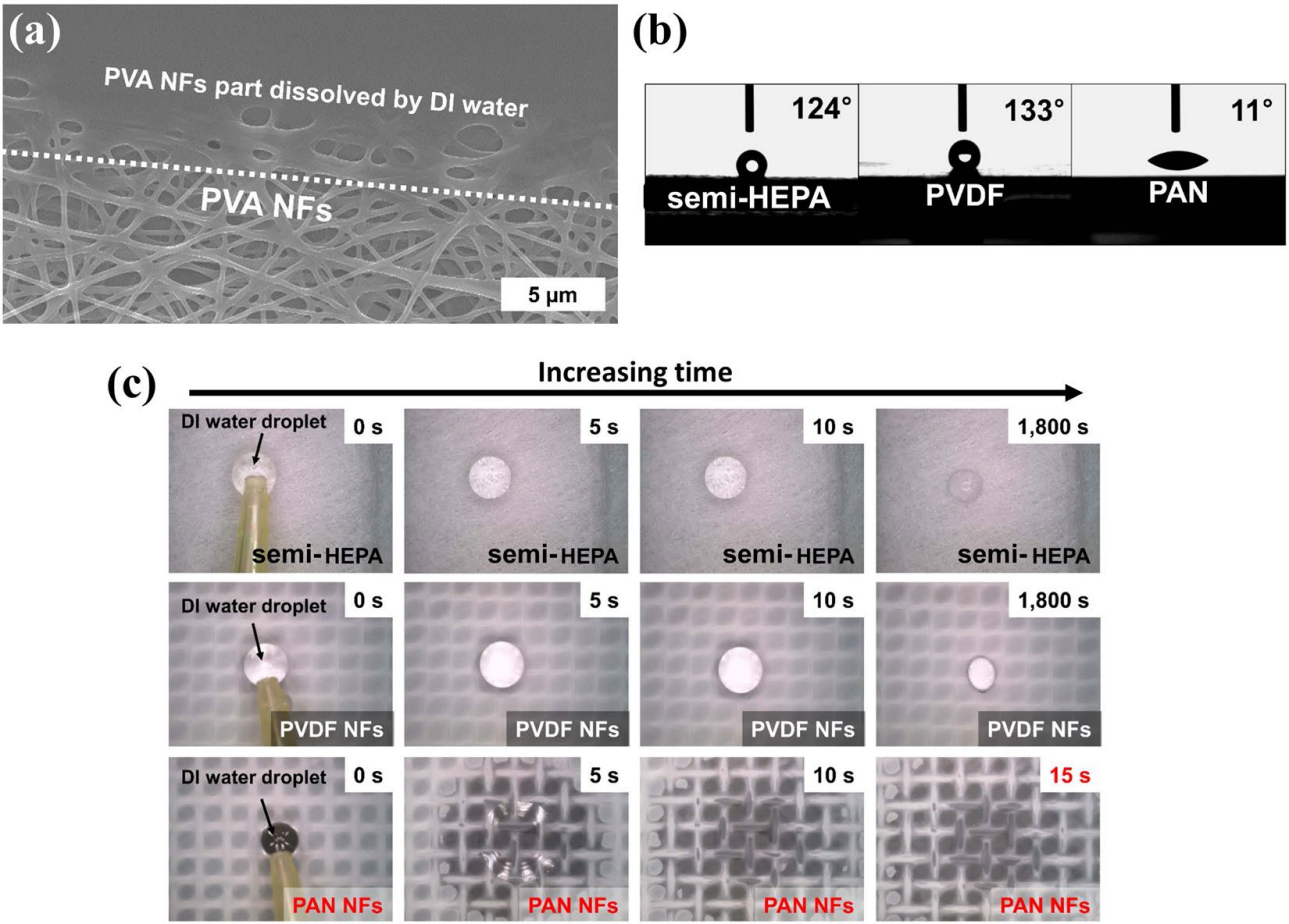
(**a**) Dissolution of PAN NFs by DI water. (**b**) Contact angle of DI water on the surface of semi-HEPA filter medium, PVDF-NF medium, and PAN-NF filter medium. (**c**) Behavior of DI water droplet on the surface of semi-HEPA filter medium, PVDF-NF filter medium, and PAN-NF filter medium.

Next, wettability tests were carried out for all filter media by measuring the contact angle (CA) of droplets of deionized (DI) water on their surface. Interestingly, the structure of the PVA-NF filter was destroyed after contact with DI water (Fig. 4(a)). By comparing the results of the DFT calculations and molecular structure analysis, it can be seen that the destruction of PVA NFs by DI water was inevitable owing to PVA’s high DPM (19.55 D, highest among the filter media examined in this study), H_2_O aggregation at the edge of the 2 nm PVA molecule, and O–H bonding from PVA, and interaction with H_2_O interaction due to high DPM. Moreover, PVA is a water-soluble synthetic polymer^34^. Figure 4(b) shows the CA of DI water on the surface of different filter media. The PAN-NF filter medium showed the lowest CA (11°) while the semi-HEPA and PVDF-NF filters showed CAs of 124° and 133°, respectively. It can thus be concluded that the PAN-NF filter medium had a hydrophilic surface. Figure 4(c) shows the behavior of the DI water droplets on the all of the filter media surfaces. The water droplets did not wet the surfaces of the semi-HEPA and PVDF-NF filters and evaporated into the atmosphere over time without absorption. On the contrary, the water droplet on the surface of the PAN-NF filter with hydrophilic characteristics was absorbed in a very short time. Again, based on the DFT calculations for DPM and the molecular structure analysis of PAN, the hydrophilic property of PAN can be explained by the steady value DPM with increasing molecular weight (see Table 1) and the uniform distribution of H_2_O in its structure (see Fig. 1(f)).

**Figure 5:**
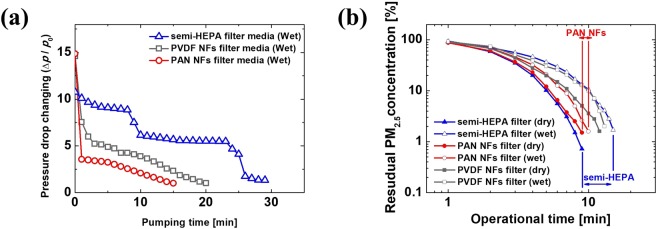
(**a**) Changes in pressure drop cross semi-HEPA filter medium, PVDF-NF filter medium, and PAN-NF filter medium as a result of water absorption. (**b**) PM_2.5_-removal performance of the semi-HEPA, PVDF-NF, and PAN-NF filter media under dry and wet conditions.

Further experiments were performed on the time-dependent pressure drop in the wet state across the commercial semi-HEPA, PAN-NF, and PVDF-NF filter media. The three filter media were stored at high-humidity (85–90% relative humidity, RH) and 30 °C for 48 h prior to the PM_2.5_-removal test to determine the effect of moisture on the air-filter media. As shown in Fig. 5(a), the pressure drop during the initial stage across the NF-based filter media in the wet state was higher than the microfiber-based semi-HEPA filter medium because more moisture was absorbed by NF-based filter media (Table 2). However, interestingly, the recovery in pressure drop across the PAN-NF filter medium was faster than the semi-HEPA and PVDF-NF filter media, which can be attributed to the hydrophilic surface and thinner thickness of the PAN-NF filter medium (in Table 2 and Fig. 4(b)). Lastly, in order to confirm the performance degradation of the filter media due to the adsorbed moisture, the PM_2.5_-removal performance in the chamber was compared with wet or dry filter media. For this, during the test, a constant voltage of DC 12 V was applied to the PM-removal device’s fan. As shown in the Fig. 5(b), the time required to remove PM in the chamber was noticeably extended by the absorption of moisture in all filter media. The performance (air filtration rate) degradation of all filter media is believed to be caused by an increase in the pressure drop across the filter media due to the adsorbed moisture. However, it is interesting to note that the performance degradation of the PAN-NF filter medium was smaller than that of the commercial semi-HEPA and PVDF-NF filter media. Eventually, the superior performance of the PAN-NF filter medium in dry and wet state was the result of its hydrophilic surface and the relatively low thickness.

## Conclusions

In summary, we demonstrated high performance of electro-spun NF-based air filter media that can effectively capture PM even in high-moisture environments. Among the filter media examined, the PAN NF filter medium showed relatively fast performance recovery under high-moisture conditions, whereas the commercial semi-HEPA filter medium showed poor recovery. The better performance of the PAN-NF filter medium originated from its structural and chemical properties and its relatively low thickness and hydrophilic surface. To recapitulate, our findings would be a strong foundation for the research and development of other NF-based devices for applications such as liquid filtration, cosmetics, drug delivery, medical textile, moisture sensing, energy storage, and air purification.

## Methods

### Preparation of polymer NF-based filter media

Polymer solutions, including polyvinylidene fluoride (PVDF; Arkema Co., France), polyvinyl alcohol (PVA; M_W_: 85,000–124,000, Aldrich, USA) and polyacrylonitrile (PAN: M_W_: 150,000, Aldrich, USA), were prepared by a sol–gel process. To fabricate the PVDF-NF filter medium, a 14 wt% PVDF solution was prepared in acetone–dimethylacetamide (acetone/dimethylacetamide ratio of 7:3 by vol/vol) and then placed in an oven at 30 °C for 12 h. PVDF NFs were deposited on the substrate by electrospinning with the PVDF solution at 50 μL/min, room temperature (25 °C), and ~29% humidity. The voltage and distance between the needles and the collector were approximately 13.5 kV and 15.5 cm, respectively. To fabricate the PVA-NF filter medium, a 7 wt% PVA solution was prepared in the oven at 95 °C for 12 h, after which the PVA NFs were deposited on the substrate by electrospinning the PVA solution at (0.5 mL/h) under the same conditions (room temperature and ~30% humidity). Here, deionized water was used as a solvent for the PVA solution. Finally, a 10 wt% PAN solution was dissolved in N,N-dimethylmethanamide at 60 °C for 12 h, and the PAN NF filter medium was prepared by electrospinning the PAN solution at 1 mL/h onto the substrate under the same spinning conditions (room temperature and ~30% humidity).

### Characterization

Field-emission scanning electron microscope (FE-SEM; Sirion FEI, USA) images were obtained at an accelerating voltage of 10 kV. Fourier transform infrared (FTIR) spectroscopy was conducted with an FTIR spectrometer (Alpha-P, Bruker Optics, Germany) from 400 to 4,000 cm^−1^. Contac angles (CAs) were measured on the surface of the filter media using a CA analyzer (Phoenix 300, Surface Electro Optics, Korea) by releasing 4.0 µL droplets of deionized (DI) water onto the surface. The behavior of the DI water droplet on the surface of each filter medium was recorded by a digital microscope imaging system (Dino-Lite Premier AD7013MZT, AnMo Electronics Corp., Taiwan) with a white light-emitting diode as the light source. The thickness of each filter medium was measured with a pair of Vernier calipers (CD-15CPS, Mitutoyo, Japan). To obtain the exact thickness, ten filter media were layered to measure the total thickness, and then the average thickness of one filter medium was calculated. The specific surface area of all filter media was measured by the BET method. For the BET measurement, an adsorption/desorption experiment with nitrogen (N_2_) gas was performed.

### Testing the performance of the air-filter media

The PM_2.5_-removal efficiency of the air filter media was calculated by comparing the PM_2.5_ concentration before and after filtration. The smoke generated by incense burning was used as a PM_2.5_ source. The size of smoke particles was measured with an optical particle sizer (OPS 3330, TSI Inc., USA). The PM_2.5_ concentration before and after filtration was monitored by laser particle sensors (PM2007, Wuhan Cubic Optoelectronic Co., Ltd., China), while the pressure drop in the filter medium was measured by a differential pressure meter (Testo 510, Testo Inc., Germany). The filtration efficiency and the pressure drop of the filter media are measured using a vacuum pump with a constant flow rate (or pumping speed) of 32 L/min. To evaluate the performance of the PM-removal device, we monitored the change in PM_2.5_ concentration in an airtight chamber while operating a fan. A DC voltage of 6–12 V was applied to the fan by a DC power supply (GPS-2303, Good Will Instrument Co., Ltd., Taiwan), and the change in the PM_2.5_ concentration was measured by an air-quality monitor (BR-SMART 126, BRAMC, China).

## Supplementary information

Moisture Effect on Particulate Matter Filtration Performance using Electro-Spun Nanofibers including Density Functional Theory Analysis
