# Supplementary material for: Moisture Effect on Particulate Matter Filtration Performance using Electro-Spun Nanofibers including Density Functional Theory Analysis
Source: Sci Rep. 2019 May 7;9:7015. doi: 10.1038/s41598-019-43127-4 (PMC6504878; doi:10.1038/s41598-019-43127-4)
Supplement: Supplementary file 1 — Moisture Effect on Particulate Matter Filtration Performance using Electro-Spun Nanofibers including Density Functional Theory Analysis [file 41598_2019_43127_MOESM1_ESM.docx]

Supplementary Information

**Moisture Effect on Particulate Matter Filtration Performance using Electro-Spun Nanofibers including Density Functional Theory Analysis**

Han-Jung Kim^1^, Seon Joo Park^2^, Dong-Ik Kim^3^, Sanghyuck Lee^2^, Oh Seok Kwon^2,4,*^, and Il Ku Kim,^5,6*^

^1^ Advanced Materials Component Research Center, Gumi Electronics & Information Technology Research Institute (GERI), Gumi 39171, South Korea

^2^ Infectious Disease Research Center, Korea Research Institute of Bioscience and Biotechnology (KRIBB), Daejeon 34141, South Korea

^3^ Center for Integrated Smart Sensors (CISS), Korea Advanced Institute of Science and Technology (KAIST), Daejeon 34141, South Korea

^4^ Nanobiotechnology and Bioinformatics (Major), University of Science & Technology (UST), 125 Gwahak-ro, Yuseong-gu, Daejeon 34141, South Korea

^5^ Institute of Integrated and Intelligent Systems, Griffith University, Brisbane, Queensland 4111, Australia

^6^ Brain Gear Incorporation, 409 Expo-ro, Yuseong-gu, Daejeon 34051, South Korea


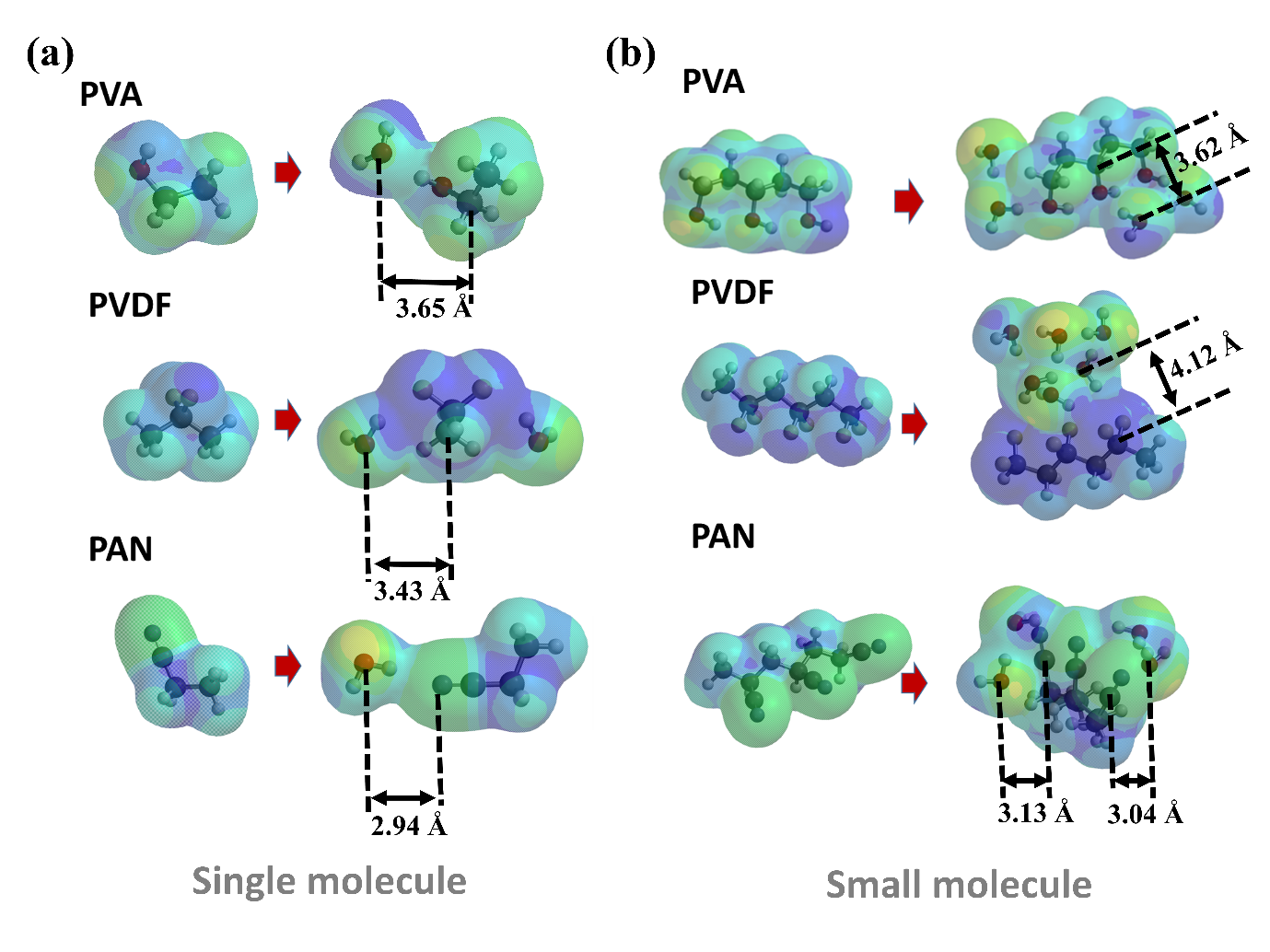


**Figure S1.** (a) DFT calculation results for single PVA, PVDF, and PAN molecules with H_2_O. (b) DFT calculation results for small PVA, PVDF, and PAN molecules with H_2_O.


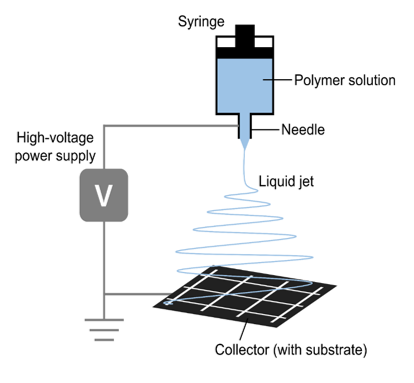

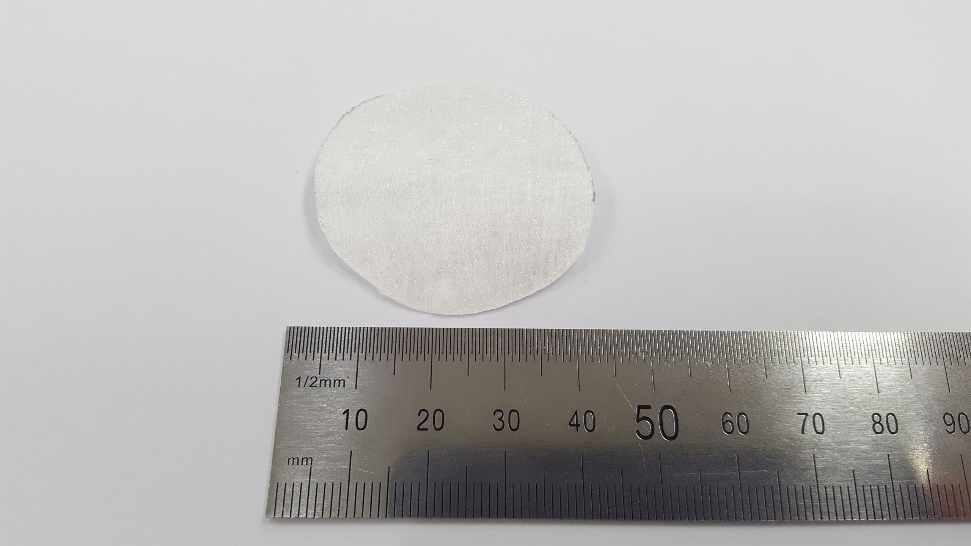


**Figure S2.** Schematic of the electrospinning process and a photo of a fabricated PAN NF-based filter medium.


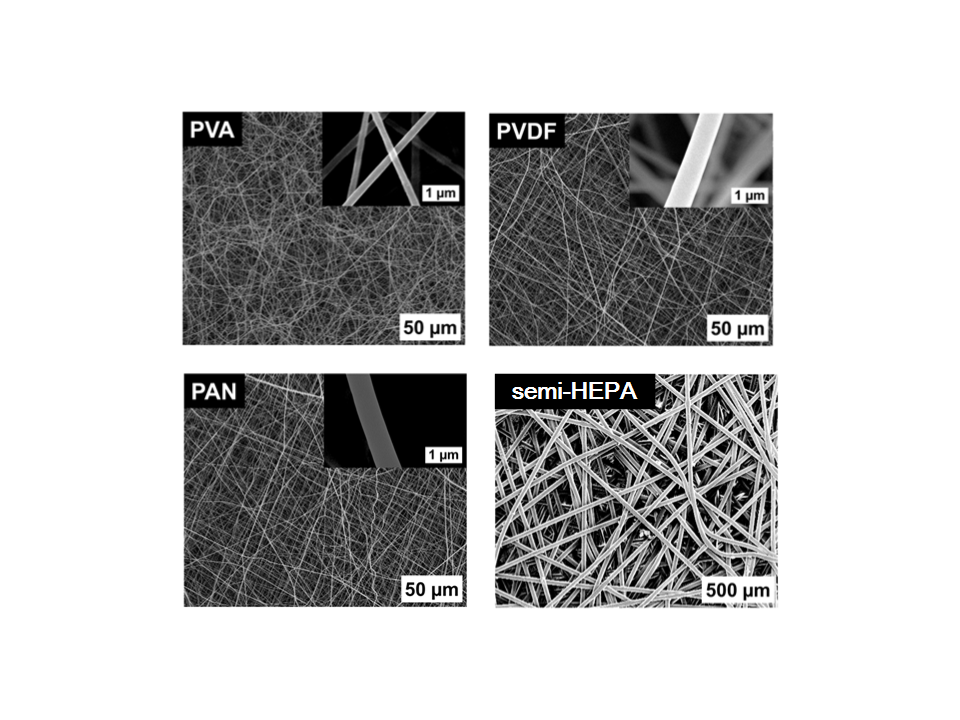


**Figure S3.** FE-SEM images of the surface of PVA-NF, PVDF-NF, and PAN-NF filters and commercial semi-HEPA filter.

We have experimentally confirmed the aggregation phenomenon of fine dust particles by humidity in a closed chamber. For this, the same amount of fine dust was injected into a chamber with humidity of 30 % and 80 % respectively. Herein, in order to supply the same amount of fine dust into the chamber, the smoke generated by completely burning the incense having a weight of 0.36 g was supplied into the chamber using the fan for a predetermined time. Then, the concentration of fine dust in the chamber was observed over time. As a result, as shown in Fig. S4, the change of the fine dust concentration inside the chamber with high humidity was relatively smaller than that in the chamber with low humidity. These experimental results can be explained by the aggregation phenomenon of fine particles by moisture. Generally, it is known that the fine dust in a dry and airtight chamber is gradually deposited inside the chamber by Gravity motion and Brownian motion.^R1-R3^ However, when the humidity is high, the fine particles absorb moisture in the atmosphere, or the fine particles aggregate with each other, and their size and weight are greatly increased, so that they are easily deposited inside the chamber.

Figure S4. Changes in concentration of PM in the chamber under different humidity conditions.

We generated the fine dust by incense burning in the closed chamber, and investigated the optical size distribution of the fine dust over time. As shown in the following additional experiment results, it was confirmed that the proportion of particles having a relatively small size (or light-weight) increases with time. These results are due to the fact that relatively large (or heavy) particles being deposited quickly in the chamber by gravity, while smaller particles are delayed due to Brownian motion.^R1-R3^

Figure S5. Optical size distribution of PM particles generated by incense burning over time.

We used the automated filter tester (8130, TSI Inc.) to measure the performance of the six filters used in this study and compare this value with the original experimental results. The test particles used were NaCl having a size of about 0.3 μm, and the test conditions were a flow rate of 32 L/min. The results are shown in the table below, which shows that the same tendency is obtained when compared with the method measured in this study. ^22,27^

Table S1. PM filtration efficiency and pressure drop of the 6-type filter measured by automated filter tester.

| Filter media | Filtration Efficiency  [%] | Pressure Drop  [mmAq] | Test Method |
| --- | --- | --- | --- |
| PVA NFs-based | 69 | 1.1 | TSI 8130  Flow Rate : 32 LPM,  Test Particle : 0.3um NaCl |
| PVDF NFs-based | 81 | 0.9 |  |
| PAN NFs-based | 92 | 1.0 |  |
| Cabin | 38 | 0.4 |  |
| Dust Mask | 84 | 2.3 |  |
| semi-HEPA | 95 | 1.2 |  |

<References>

R1. Kim, H.-J., Kim, D.-I., Kim, S.-S., Kim, Y.-Y., Park, S.-E., Choi, G., Lee, D. W. & Kim, Y. Observation of convection phenomenon by high-performance transparent heater based on Pt-decorated Ni micromesh. *AIP Adv.* **7**, 025112 (2017).

R2. Nazaroff, W. Indoor particle dynamics. *Indoor Air* **14**, 175-183 (2004).

R3. Hussein, T., Hruška, A., Dohányosová, P., Džumbová, L., Hemerka, J., Kulmala, M. & Smolík, J. Deposition rates on smooth surfaces and coagulation of aerosol particles inside a test chamber. *Atmos. Environ.* **43**, 905-914 (2009).
